# β-Confident-registry: aiming to be largest-ever studied cohort of cryopyrin-associated periodic syndromes (CAPS) patients. Study design and baseline characteristics

**DOI:** 10.1186/1546-0096-9-S1-P16

**Published:** 2011-09-14

**Authors:** J Kuemmerle-Deschner, D Rothenbacher, U Walker, H Tilson, H Hoffman, P Hawkins

**Affiliations:** 1 Division of Pediatric Rheumatology, University Hospital Tuebingen, Tuebingen, Germany; 2 Novartis Pharma AG, Basel, Switzerland; 3 Rheumatologische Universitäts-Poliklinik, Basel, Switzerland; 4 University of North Carolina, Chapel Hill, NC, USA; 5 Division of Rheumatology and Allergy/Immunology, University of California at San Diego, USA; 6 Department of Medicine, University College London Medical School, London, UK

## Background

Clinical data, especially long-term follow-up, is limited in CAPS due to its extreme rarity. We report here the design of a global observational registry for canakinumab (Ilaris®). The registry is monitored by an external steering committee with expertise in auto-inflammatory disease and registry design.

## Aim

The primary objective is to monitor overall safety of routine care with canakinumab in a large cohort (N≥400) of CAPS patients. Secondary objectives include exploring growth and development patterns in children and to measure the long-term impact of canakinumab on disease progression.

## Methods

CAPS patients receiving canakinumab as part of their routine care are included in this study for a minimum of 5-years follow-up. Data from routine clinic assessments is supplied at 6-monthly intervals via a web-based application. Selected safety events potentially associated with anti-IL-1 therapy such as serious infections, malignancies, hypersensitivity and disease activity/progression is analyzed. Signs and symptoms of systemic inflammation, neurologic and ophthalmologic status and the potential for canakinumab therapy to prevent amyloidosis and to ameliorate sensorineural deafness are assessed. Patterns of growth/development, pregnancies, outcomes of vaccination, dosing pattern and tolerability are ascertained. All patients are followed until the registry ends.

## Results

Baseline data from the first 60 CAPS patients, including 15 pediatric patients (age <18 years), enrolled to date, are presented in table 1.

**Table 1:** 

	FCAS (n=10)	MWS (n=37)	NOMID (n=6)	Other (n-5)
Age <4 years	0	2	1	1

Age 4- <18 years	2	6	3	1

Age ≥18 years	8	29	3	3

Male, n (%)	3 (30.0)	20 (54.1)	3 (50.0)	2 (40.0)

NLRP3 mutation, n (%)	10 (100.0)	35 (94.6)	5 (83.3)	2 (86.7)

Disease duration (months, mean)	550	370	172	64

Rash/arthralgia/headache/conjunctivitis (%)	100/100/50/80	97.3/97.3/78.4/75.7	100/100/93.4/66.6	80/100/20/40

History of anaemia (%)	10	8.1	50	0

Prior SAA value (mean, mg/L)	37	26	47	3

Prior IL-1 inhibitor treatment, n (%)	0	14 (37.8)	1 (16.1)	2 (40)

## Conclusion

Baseline characteristics of the 60 patients enrolled to date demonstrate an expected disease background. Upon availability of a larger data-set, subanalyses will yield valuable insights into disease characteristics and modification properties of canakinumab.

